# Thoracic Endometriosis Syndrome: A Comprehensive Review and Multidisciplinary Approach to Management

**DOI:** 10.3390/jcm13247602

**Published:** 2024-12-13

**Authors:** Camran Nezhat, Nikki Amirlatifi, Zahra Najmi, Angie Tsuei

**Affiliations:** Center For Special Minimally Invasive and Robotic Surgery, Camran Nezhat Institute, Woodside, CA 94061, USA; nikki.amirlatifi@camrannezhatinstitute.com (N.A.); zahranaj@yahoo.com (Z.N.); angie.tsuei@camrannezhatinstitute.com (A.T.)

**Keywords:** thoracic endometriosis, diaphragmatic endometriosis, catamenial pneumothorax, catamenial hemothorax, catamenial hemoptysis, lung nodules endometriosis, pulmonary endometriosis, thoracic endometriosis syndrome, endometriosis of the lung, MIS, robotic surgery

## Abstract

**Background:** Endometriosis is a systemic, inflammatory, estrogen-dependent condition characterized by endometrial stroma and gland-like lesions outside of the uterus. It causes a range of symptoms, notably chronic pelvic pain, infertility and organ dysfunction. Thoracic endometriosis syndrome (TES) has been described as endometriosis that is found in the lung parenchyma, pleura and diaphragm. It may be asymptomatic or present with symptoms of catamenial pneumothorax, hemothorax, hemoptysis, isolated chest pain, shoulder pain or findings of lung nodules. **Aim:** The aim of this review is to provide a comprehensive overview of thoracic endometriosis syndrome (TES), including its clinical presentation, diagnostic challenges, and current management strategies. This review aims to highlight the importance of a multidisciplinary approach in the treatment of TES, emphasizing conservative management and the role of minimally invasive surgical techniques for refractory cases. **Conclusions:** Thoracic endometriosis syndrome appears to be a marker of severe endometriosis. As much as possible, the patient with TES is managed conservatively, with surgery reserved for refractory cases. When surgery is recommended, the procedure is conducted through a multidisciplinary minimally invasive approach, with video-assisted thoracoscopic surgery (VATS) and video-assisted laparoscopy. Meticulous intraoperative survey, the removal of endometriosis implants with and without robotic assistance and post-operative hormonal therapy may be recommended to prevent recurrence.

## 1. Introduction

Endometriosis is a challenging chronic disease, with a 10% cited prevalence in the population [1]. It is described as the presence of endometrial-like glands and stroma outside of the endometrium [2,3,4], causing a range of symptoms, notably chronic pelvic pain, infertility, and organ dysfunction [5]. A sizable number of extra abdominopelvic manifestations have been described, more commonly in the thorax [6,7,8,9,10]. Schwarz first described endometriosis in the lung in 1938 [11]. Barnes then described a case of thoracic endometriosis syndrome (TES) in 1953 in a patient with hemothorax [12]. Shortly after, in 1958, Maurer et al. described the surgical management of diaphragmatic endometriosis [13]. Since then, rising awareness has led to many case reports and small studies being published. However, due to the relatively low prevalence of TES, larger studies have been difficult to produce, and there is a vast heterogeneity in the definition, diagnosis and management of this disease.

Thoracic endometriosis syndrome has various presentations. The most commonly described symptom is catamenial pneumothorax, which occurs 24 h prior or within 72 h after the onset of menses [14]. Less common variations include hemothorax, hemoptysis and pulmonary nodules. A widening of the definition of TES to include endometriosis-related diaphragmatic rupture (hernia), catamenial chest pain and recurrent pleural effusion has been proposed [15]. This expanded criterion has already been used to successfully detect more cases [16].

It is reported that up to 80% of patients with TES have concurrent pelvic endometriosis [6,17]. The reported age at diagnosis of pelvic endometriosis is between 25 and 35 years [18]. Pelvic endometriosis is thought to develop 5–7 years prior to symptoms of TES [6,10]. TES is generally found in older patients, with a mean age of 35 at diagnosis [6,10,16,19,20,21]. It is still unclear whether it is a late presentation of endometriosis or the result of a delay in diagnosis. Perhaps, it is a marker of endometriosis progression rather than a manifestation of it [6,22,23]. Risk factors specific to TES have been hard to establish, with some citing an association with a history of previous pelvic surgery [24,25,26]. It can take multiple exacerbations to receive a correct diagnosis [10,27]. Thus, the aim of this review is to provide a comprehensive overview of thoracic endometriosis syndrome (TES), including its clinical presentation, diagnostic challenges, and current management strategies. This review aims to highlight the importance of a multidisciplinary approach in the treatment of TES, emphasizing conservative management and the role of minimally invasive surgical techniques for refractory cases.

## 2. Etiology

Many theories have been postulated regarding the etiology of extra abdominopelvic endometriosis. No single theory fully explains the various clinical manifestations of TES. Endometriosis is likely the result of a complex multi-factorial phenomenon that involves initiating, propagating and predisposing factors, which are in turn influenced by genetic, epigenetic, immune, hormonal and environmental components [28].

### 2.1. Retrograde Menstruation Theory

Sampson’s theory of retrograde menstruation [29], which may have been preceded by Schron’s noted observations centuries earlier [30], is currently the most widely accepted theory [31]. It is postulated that during menstruation, a number of endometrial cells move through the fallopian tubes and into the peritoneal cavity. In the presence of the right genetic, hormonal and microenvironmental factors, these cells are free to implant on any desired peritoneal surface. The correct setting may involve factors that prevent the clearance of ectopic lesions, allow for peritoneum remodeling and offer an altered peritoneal fluid composition [28]. Once in the peritoneal cavity, the endometrial cells follow the clockwise peritoneal currents, which flow from the pelvis to the right paracolic gutter, then finally towards the right hemidiaphragm [31]. The falciform and phrenicolic ligaments interrupt the flow to the left hemidiaphragm. Once in the subdiaphragmatic area, the endometrial cells either implant on the diaphragm or undergo transperitoneal/transdiaphragmatic migration to the pleural cavity via congenital or acquired fenestrations within the diaphragm [32]. In support of this theory is the fact that endometriosis is found 9 times more frequently on the right side than on the left [6,10]. Praetorius et al. have conducted recent molecular analysis studies that suggest endometriosis as an oligoclonal disease with dissemination due to multiple epithelial clones traveling together [33]. Recent DNA sequencing studies that support retrograde menstruation suggest the presence of oligoclones of epigenetically defective endometrial stromal cells and epithelial cells that carry cancer-driving mutations [34].

### 2.2. Coelomic Metaplasia

In 1924, Robert Meyer hypothesized that endometriosis may arise from coelomic epithelium [35,36]. It is known that the endometrium and the pleural and peritoneal mesothelium share the same embryonic origin. It is postulated that mesothelial cells have the potential to transform into endometrial glands and stroma, via the process of metaplasia. This process is thought to be influenced by hormonal and immunological factors. This theory may explain the presence of endometriosis in pre-pubertal girls [37] and the formation of ovarian endometriomas [38]. It does not, however, explain the preferred laterality of thoracic endometriosis, nor the intrapulmonary cases found.

### 2.3. Lymphatic and Hematogenous Dissemination Theory

The theory of “benign metastases”, first published by Halban in 1925, hypothesizes that endometrial cells are carried through lymphatic and hematogenous channels via microembolization [39]. Lattes et al. postulated in 1956 that trauma, or the manipulation of uterine tissue through procedures, could predispose endometrial tissue to enter the lymphovascular circulation [40]. This could explain the presence of endometriosis in distant sites, and the bilaterality in the bronchopulmonary tree. Recently, Samani et al. demonstrated that all of their immunocompetent mouse models exhibited disseminated endometriosis in multiple organs after being inoculated with endometrial tissue in the peritoneum. It is thought that widespread micro metastasis is more common than expected. It may go clinically unrecognized and may contribute to diffuse non-specific manifestations of the disease [41].

### 2.4. Genetic Epigenetic Theory

More recently, alternative hypotheses have been promulgated and include, but are not limited to, a genetic/epigenetic theory, circulating stem cells and progenitor cells theory, repeated tissue injury and repair caused by uterine hyperperistalsis theory, and a fetal or adolescent origin [42].

## 3. Clinical Presentation

Thoracic endometriosis syndrome has a wide range of manifestations, with up to 70% patients who remain asymptomatic [43]. Without a high index of suspicion, the diagnosis can be delayed [8]. Among symptomatic patients, catamenial pneumothorax (72%) is the most common presentation. Other variations include catamenial hemothorax (13%), catamenial hemoptysis (10%), and rarely pulmonary nodules (4%) [31]. Classically, symptoms have a temporal relationship to menses, but recent data show that this is not always the case [44,45,46,47]. Although less common, catamenial symptoms may also present in a non-catamenial fashion [48]. The term thoracic endometriosis related pneumothorax (*TERP)* has recently been used to alleviate this confusion but has not yet been widely adopted [46,49].

The right side of the thoracic cavity is involved in 90% of all manifestations, except for lung nodules [10]. Bilateral pathology proven lesions are rare, but have been reported [30]. Patients may present with acute symptoms; however, the adequate control of the disease relies on the recognition and treatment of the underlying chronic condition. An interval of almost 19 months is reported between the first episode of pneumothorax and the diagnosis of TES [17]. Key findings in the patient’s history may include a relationship with menses, predominantly right-sided symptoms, reproductive age, the presence of recurrent disease and a history of infertility [22].

Symptoms of TES are largely related to the locations of the lesions, and can present within the visceral and parietal pleura, the lung parenchyma, the tracheobronchial tree, and the diaphragm [10].

Tsuboshima et al. recently postulated a theory about the higher recurrence rate of pneumothoraxes in patients with TES and concurrent pelvic endometriosis. They began from the belief that symptomatic endometrial lesions (SELs) and asymptomatic endometrial lesions (ASELs) often coexist. They believe that endometrial tissue has a variable potential for proliferation and invasiveness, and that pneumothorax is the result of two types of disease progression: Type A and Type B. Both types start with asymptomatic endometrial lesions in the pelvis that migrate to the lung, and can rupture the visceral pleura, turning into a symptomatic endometrial lesion and causing a pneumothorax. Type A, which involves more aggressive dissemination, causes the transformation of the ASELs into SELs in the pelvis as well, which cause more propagation to the lung and create more opportunities for ASELs to become SELs in the lung. Type A progression has a higher propensity for proliferation and invasion; therefore, even after surgery, residual ASELs that are left behind may lead to more recurrences. Type B has slow proliferation with ASELs in the pelvis (a subclinical condition of pelvic endometriosis) [50].

### 3.1. Pleural Thoracic Endometriosis Syndrome

Pleural symptoms are described as pleuritic chest pain, shoulder pain, cough and shortness of breath. Symptoms are often indistinguishable from a spontaneous pneumothorax. It is estimated that up to 30% of women referred for surgical treatment of spontaneous pneumothorax have thoracic endometriosis [26].

Four theories have been postulated regarding the pathophysiology of catamenial pneumothorax [51], including the spontaneous rupture of blebs; the loss of cervical mucus during menses causing a transdiaphragmatic air shift from the genital tract due to negative intrathoracic pressure [13] (of note, this cannot explain why pneumothorax can occur in patients who underwent hysterectomy [52]; sloughing of endometrial implants from visceral pleura with subsequent air leakage [51]; prostaglandin F2-alpha, a potent constrictor of bronchioles and a vasoconstrictor, becomes elevated in menstruating women’s plasma, and may lead to alveolar rupture. The swelling of pulmonary endometrial implants in terminal bronchioles may cause localized hyperinflation and cause pneumothorax by a check-valve mechanism [53,54]. Of note, this cannot explain the tendency towards right-sided lesions.

Catamenial hemothorax will present with non-specific symptoms such as cough, shortness of breath, and pleuritic chest pain. The signs may mimic those of pulmonary embolism [55].

### 3.2. Bronchopulmonary and Parenchymal Thoracic Endometriosis Syndrome

Bronchopulmonary TES is a less common variant, and may present as mild to moderate catamenial hemoptysis or as a rare lung nodule identified on imaging. Catamenial hemoptysis is noted in younger patients and is rarely life-threatening. It may initially be confused with tuberculosis [20]. Lung nodules are most often incidental findings found in older women and in the periphery of the lung [10].

### 3.3. Diaphragmatic Thoracic Endometriosis Syndrome

Diaphragmatic endometriosis is most often asymptomatic [4]. Symptoms may arise due to phrenic nerve irritation and referred pain to the periscapular region or to the neck, most often on the right side [6]. Isolated diaphragmatic endometriosis may present with cyclic neck, shoulder, right upper quadrant or epigastric pain [56,57,58]. It is thought that cyclical necrosis of endometrial implants on the diaphragm leads to diaphragmatic defects that may eventually coalesce into larger defects that can lead to hernia [46]. Rarely, diaphragmatic rupture may be the presenting sign of thoracic endometriosis [59].

## 4. Diagnosis

### 4.1. History

The suspicion of TES is primarily based on the patient’s history, which is sufficiently sensitive to recommend surgical intervention when appropriate [8]. Concurrent pelvic endometriosis is likely, and patients who present with TES symptoms should also be questioned regarding endometriosis symptoms; dysmenorrhea, dyspareunia, dysuria, dyschezia, infertility, and pelvic pain. The use of the free endometriosis risk advisor app as a non-invasive screening test may be a helpful adjunct to diagnosis [60].

### 4.2. Imaging

A CT scan or chest X-ray is generally performed for the diagnosis of catamenial pneumothorax. The pneumothorax can be of any size and may result in a shifting of the mediastinum [61]. Radiological features associated with TES, which may also be seen on CT, include pneumomediastinum, pneumoperitoneum, ground glass opacities, bronchial wall thickening, thin-walled cavities within the lung parenchyma, and bullous formation [62]. Ultimately, CT shows low specificity for the identification of endometriosis, and is instead used to rule out other underlying intrathoracic disease [62].

A chest and abdomen MRI is considered the most appropriate pre-operative imaging study at this time [17]. The reported sensitivity for diaphragmatic endometriosis by MRI is 78–83% when using fat-suppressed T1-weighted sequences [63]. The “air filled bubble” sign on MRI may be indicative of small diaphragmatic perforations [62]. Separate imaging at the time of menses and comparison to mid-cycle may help visualize discrepancies, as previously documented findings may disappear, increasing suspicion of TES [64].

### 4.3. Bronchial Biopsy and Brush Cytology

Bronchial biopsy, thoracoscopic biopsies and cytological washings have been of limited use. Endometrial implants tend to lie on the peripheries of large bronchi, rather than on the mucosa, and are often missed [24]. Brush cytology has been useful in cases of bronchopulmonary endometriosis [65].

### 4.4. Video-Assisted Thoracoscopy (VATS)

Video-assisted thoracoscopy is the surgery of choice in patients with catamenial pneumothorax, as it is diagnostic and therapeutic [14,27,58,66,67]. In a recent review by Andres et al., 628 patients with TES were evaluated during VATS and were found to have endometrial implants most commonly on the diaphragm (78.82%), on the pleura (14.33%), in the lungs (4.46%), and in all three locations (1.11%) [9].

### 4.5. Video Laparoscopy

All video-laparoscopic evaluations for pelvic endometriosis should include the inspection of the diaphragm for endometriotic lesions [45]. The presence of “sentinel lesions”, small superficial endometriotic implants, less than 1 cm in diameter on the right anterior surface of the diaphragm, is suspicious for the presence of additional posterior implants, which are generally more likely to be symptomatic [58,68,69]. Lesions on the diaphragm tend to be associated with more severe pelvic endometriosis [21,45,68,70]. The reported rate of diaphragmatic endometriosis is 0.19–1.5% in patients with pelvic endometriosis. Of note, Pagano et al. recently reported a rate of 4.7% diaphragmatic endometriosis in their patients, after complete laparoscopic inspection of the diaphragm was performed in patients’ undergoing laparoscopy for endometriosis at their tertiary care referral center [70].

### 4.6. Intraoperative Markers

Various methods have been used to improve the visualization of the endometriosis implants intra-operatively. Staining agents such as 5-aminovulinic acid fluorescence, indigo carmine, methylene blue, indocyanine green, and peritoneal fluid painting have been reported with some success, but data are sparse on a preferred technique at this time [71]. There may be a role for the preoperative localization of small or difficult to reach endometriotic lesions by means of metallic materials (hook wire, microcoil or spiral coil), dyes (methylene blue or indigo carmine), contrast agents (lipidiol, iodine or contrast agents) or radiotracers (technetium-88 m) to improve visualization and decrease operative time during VATS. Lee et al. have shown preliminary success, but they have not yet established a protocol [72].

### 4.7. Histology and Immunohistochemistry

The histological diagnosis of thoracic endometriosis is performed via the identification of endometrial-like glands, stroma and hemosiderin-laden macrophages on hematoxylin and eosin stains (H&E) [73]. In many cases, the diagnosis is challenging due to scant tissue samples, fibrosis, inflammation, and the effect of thermocoagulation. Often, only small foci of endometrial stroma are found in pleural tissues. Alifano et al., in 2007, suggested that if only endometrial stroma is present in a TE sample, the diagnosis of thoracic endometriosis is “probable”. As such, there has been a push to demonstrate the hormone-dependent nature of stromal spindle cells by immunohistochemistry, and perhaps improve the classification and detection of the disease [42].

Mecha et al. recently examined the role of stromal endometriosis in the diagnosis of thoracic endometriosis syndrome. Immunohistochemistry evaluations were done with antibodies specific for estrogen (ER), progesterone (PR) and CD10. They found that stromal endometriosis was diagnosed in 52% of the immunohistochemical studies as opposed to 10% of the histological studies of TES. The presence of CD10 alone in specimens is enough to indicate stromal endometriosis, and in the presence of ER/PR it can help identify catamenial pneumothorax [74]. They suggest a mandatory reporting of stromal endometriosis in all specimen samples. Another study proposes the use of an “aggregated pattern” of positive ER, PR and CD10 staining on immunohistochemistry to help identify TES cases with a high chance of recurrence. Markers such as IFITM1 and PAX8 have demonstrated a high sensitivity in the identification of ectopic endometrial cells, and limited studies have shown promising data [42].

TES diagnosis should therefore include a pathologist who is familiar with endometriosis [48]. Ghigna et al. proposed that every pleural/diaphragm/lung or bronchial biopsy should be evaluated for histology, and in the setting of non-descript findings in a patient with a clinical background suspicious for TES, immunohistochemistry should be performed [75].

### 4.8. Biomarkers

Cancer antigen-125 has been evaluated as a serum marker for catamenial pneumothorax. Bagan et al. reported a high probability of endometriosis with CA-125 levels of 76 U/mL and higher [76]. Unfortunately, CA-125 is non-specific and can be raised in any process that irritates mesothelial cells. Kiss et al. have recently identified circulating endometrial cells in the peripheral blood of patients with spontaneous pneumothorax that is suspicious for catamenial pneumothorax. Circulating endometrial cells exhibit cytomorphological changes based on hormone levels and differences in their gene expression, and in the future, could play a role in identifying endometriosis in patients with spontaneous pneumothorax [77,78].

## 5. Management

**Figure 1:**
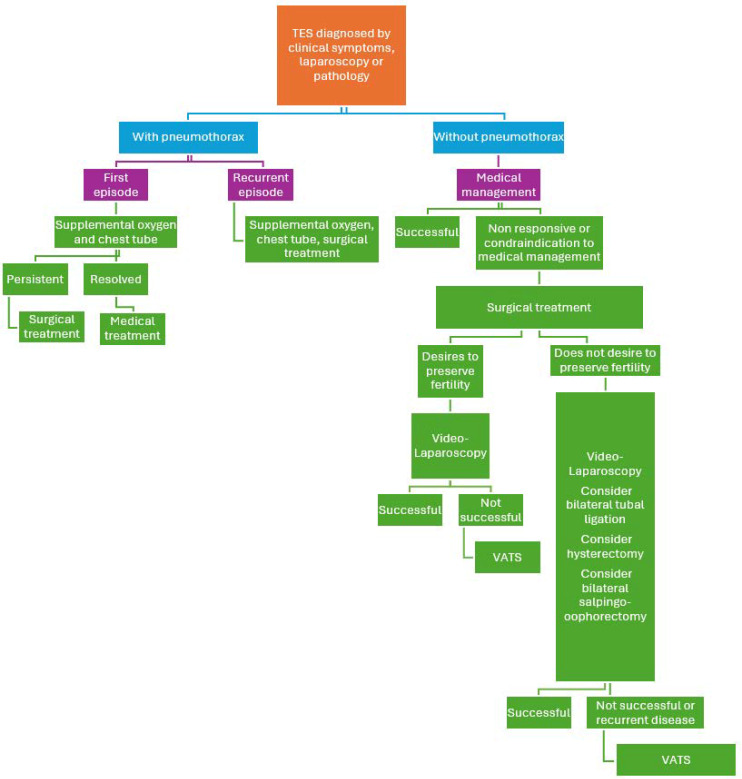
Options for management with TES.

The management of TES can be different in patients with and without pneumothorax (see Figure 1). Thoracic endometriosis syndrome management involves medical hormonal therapy as much as possible. Options for treatment include GnRh analogs, oral contraceptives, progestins, aromatase inhibitors and GnRh antagonists, as we suggest in pelvic endometriosis. Danazol is no longer recommended as a first-line therapy due to its bothersome side effect profile, such as acne, edema, vaginal spotting, weight gain, muscle cramps, deepening of voice, and increase in facial hair [79]. Aromatase inhibitors have also been used, but no data are yet available. GnRh antagonists can be given orally, but no data are available yet on its effectiveness in TES [25]. Failure is common, with recurrence as the primary indication for invasive management [22]. Patients who do not respond to medical management or have contraindications to medical management need to be treated surgically. In patients who present with pneumothorax, the first step is to stabilize the patient. Tube thoracostomy or needle thoracentesis are used for the acute treatment of pneumothorax, pleural effusions and hemothorax [80]. In cases of persistent pneumothorax and recurrent episodes, patients need to be managed surgically. The surgical treatment of choice is VATS with concurrent video laparoscopy.

In patients who are candidates for VATS, simultaneous video-laparoscopic surgery may be recommended. In our 2014 retrospective observational study of 25 patients, we reported that 76% of patients with TES had both pleural and abdominal diaphragm involvement [8]. Diaphragmatic exploration is considered incomplete if only one face of the diaphragm is inspected [81]. Both gynecology and thoracic surgery evaluation are recommended, and a chest and abdomen MRI is recommended [21]. The goal of surgery is the excision of all visible endometriotic foci in the thorax and pelvis [22]. Consent for sterilization may be obtained in applicable patients. Older patients who no longer desire fertility conservation may elect to undergo bilateral salpingo-oophorectomy with or without hysterectomy prior to attempting concomitant procedure. Even after bilateral salpingo-oophorectomy, dormant endometrial implants may activate with exogenous estrogen administration [17,82].

### 5.1. VATS

The patient should have surgery scheduled within the first few days of menses to allow increased diagnostic yield [27]. Intraoperatively, diaphragmatic involvement is managed by the size and location of the lesion. The lung is inspected for blebs or bulla, and apical resection and apical pleurectomy are performed [21]. Specific sites, such as visceral pleura of S4 and the parietal pleura of the sixth intercostal space, should be inspected for lesions [83]. Superficial endometriotic implants may be excised or fulgurated by bipolar diathermy, CO_2_ laser, Nd-YAG laser, argon laser, or plasma energy [22]. Deeper endometriotic lesions are sharply dissected, with resection and repair with an endoscopic stapler device or suture. For larger resections, a mini-thoracotomy is recommended to allow repair with X-shaped stitches [46]. Concomitant bullous dystrophy is resected. Deep parenchymal nodules require pulmonary wedge resection with an endoscopic stapler device. Deep diaphragmatic lesions require diaphragmatic resection by use of an endoscopic stapler device or by excision and manual suturing. Parietal pleura involvement requires partial pleurectomy. The phrenic nerve, pericardium and superior vena cava must be clearly identified and avoided [8].

Pleurodesis, the obliteration of pleural space via adhesion formation, can be done mechanically or chemically, and allows for a reduction in spontaneous pneumothorax recurrence [84]. Chemical pleurodesis is obtained with talc [21]. Mechanical pleurodesis, which may have a higher recurrence rate, is done through pleural abrasion and partial pleurectomy after confirming the absence of any residual endometrial implants [21]. The use of pleurodesis effectively reduces the recurrence rate of catamenial pneumothorax by 20–25% [58]. Pleurodesis is used judiciously, as the iatrogenic adhesion formation may make repeat VATS more dangerous. In 2014, we reported a retrospective observational study wherein 25 patients underwent VATS with video laparoscopy for thoracic, diaphragmatic, and abdominopelvic endometriosis without the use of pleurodesis, and no reports of recurrent pneumothorax were found in the 3-month to 18-month follow-up period [8].

Chiantera et al. reported a retrospective study of laparoscopic “en bloc” right diaphragmatic peritonectomy according to the Sugarbaker technique that they performed on nine women, which appeared to be highly effective in reducing symptomatic diaphragmatic endometriosis cases, with no major intraoperative or postoperative complications [85].

Rarely, complications such as an acquired diaphragmatic hernia may be seen post-operatively. Unintended transmural injury or a small tear in the diaphragm may inadvertently expand due to the pressure gradient between the thorax and abdomen, and abdominal organs may herniate into the thorax [69]. A synthetic mesh is used to close larger diaphragmatic defects [86,87]. Although rare, there is a risk of diaphragm paralysis via phrenic nerve injury, which is more commonly unilateral and found on the left side [88].

The use of suction prior to suture closure may drain excess fluid from dependent parts of the lung and limit the need for a chest tube post-operatively [89]. Exploratory thoracotomy now plays a limited role, reserved for cases of failure of prior thoracoscopic exploration [47] and in complicated diaphragmatic lesions, in order to avoid important structures [90].

### 5.2. Video Laparoscopy

Concomitant pelvic endometriosis is frequent, and video laparoscopy at time of VATS is recommended [8,22]. In our experience and following recent recommendations, we suggest to start with video laparoscopy because it is less invasive and less painful, as it does not require the placement of a chest tube. The goal of surgical management is ablation, excision and resection of endometriosis. It is unclear whether incidental diaphragmatic endometriosis findings should be treated. In some cases, they may lead to infiltrative disease, which may be more difficult to treat, have increased need for pain control and show a higher recurrence rate [45]. The removal of incidental findings is only recommended if the following pertains: (1) the surgeon is sufficiently experienced with the treatment of deeply infiltrating endometriosis; (2) the endometriotic lesions are superficial and located a safe distance from important structures; (3) a thoracic surgeon is available; (4) the patient has received adequate preoperative counseling involving the risks of such a procedure [45].

**Figure 2:**
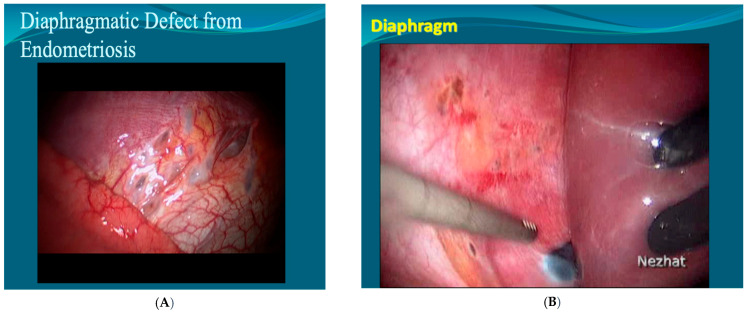
Diaphragmatic defects from endometriosis: (**A**) Superficial endometriosis and diaphragmatic defect. The figure shows superficial endometriosis and diaphragmatic defect; (**B**) Deep infiltrative endometriosis. The figure shows deep infiltrative endometriosis of the diaphragm.

The successful diagnosis and treatment of endometriosis of the diaphragm will depend on the complete visualization of the diaphragm. In order to maximize visualization, an adjustable-viewing-angle endoscope should be used, and the patient should be placed in a steep reverse Trendelenburg position. Atraumatic liver retractors should be used to push the liver caudally [8]. A 0° video-laparoscope, followed by a secondary survey using a systematic approach with a 30° video-laparoscope, can increase the detection of diaphragmatic lesions [91]. Alternatively, the use of an angled endoscope with a rod–lens system with variable directions of view may be helpful in difficult-to-reach areas (Figure 2B) [92]. In some cases, cutting the falciform ligament is necessary to allow for the complete evaluation of the right hemidiaphragm [44]. Endometrial implants are often visualized as black, blue, or reddish-purple lesions; however, they may appear as clear vesicular lesions, fibrotic white lesions, and liver adhesions as well [44].

Abdominopelvic and visceral superficial diaphragmatic endometriosis is treated via hydro-dissection followed by the excision or CO_2_ laser vaporization (Figure 2A) [45]. CO_2_ laser is preferred over electrocautery as it offers more precise treatment with less depth of penetration and less thermal spread [6]. Alternatives include plasma jet energy and ultrasonic energy. Neutral argon plasma can also be used without any thermal effect on the endometriotic biopsies [93]. The use of monopolar energy is discouraged due to the proximity to the heart and the risk of cardiac arrhythmias, as well as potential muscular contractions [8].

Small diaphragmatic perforations can be reapproximated with an endoscopic stapler device [46,58] or sutured with non-absorbable sutures. However, for large perforations, manual suturing and the addition of mesh are recommended [22]. When the full thickness of the diaphragm is involved, VATS will aid in achieving complete resection. The use of robotics may help navigation and allow for more precise excision and suture re-approximation [89]. The robotic approach allows for more comfortable suturing in hard-to-reach areas. At times, the robotic cart may be cumbersome in the setting of a multi-disciplinary approach [89].

The resection of bowel endometriosis may be limited during a concurrent procedure due to the theoretical risk of peritonitis expanding into the mediastinum [89]. An individualized approach is recommended in cases of extensive abdominopelvic endometriosis. Furthermore, concurrent tubal ligation has been suggested in patients desiring sterilization undergoing laparoscopy [51]. The addition of this step may decrease the air shift from the pelvis into the peritoneum, and in theory, may decrease catamenial pneumothorax in patients affected by this mode of spreading [44,51].

There is always a risk of CO_2_ pneumothorax secondary to the perforation of the diaphragm. This must be followed by the immediate insertion of a thoracic tube to evacuate the iatrogenic pneumothorax [45]. The use of liver retractors may cause liver injury, and additional port sites may inadvertently disrupt the diaphragm as well [43].

## 6. Prognosis and Follow Up

Andres et al. reported a recurrence of pneumothorax after surgical treatment of 29% within a 3- to 168-month follow-up period [9]. Discrepancies in the recurrence rates are due to the small study sizes and variable follow-up lengths [94]. Postoperatively, a treatment plan should be formulated to induce amenorrhea for all patients with TES. The goal is to suppress ovarian hormone production in order to prevent the reseeding of the thorax by pelvic endometriosis. No hormonal treatment has shown superiority, and ultimately it is the patient’s choice [95]. Factors that may play a role in decision-making include the cost of the medication, the side effect profile, availability, and the treatment goals [71]. The desire for childbearing must be addressed.

In a recent study by Naem et al., only 21% of the patients’ who underwent surgical management of symptomatic diaphragmatic endometriosis complied with the recommended post-operative hormonal therapy [96]. They believe that their low compliance may be attributed to a high infertility rate, a desire to conceive, a higher degree of pre-operative pain, persistence of post-operative pain and possible lower satisfaction with the operation [96]. Soriano et al. found the presence of infertility to be notably higher in patients with TES [23]. In their study of seven patients with TES, they found severe concomitant pelvic disease, and referred all patients that aspired to childbearing for IVF or adoption [23]. Ottolina found conflicting evidence in their cohort, and although they reported a high rate of severe pelvic endometriosis in their patients with catamenial pneumothorax, a minimal effect on fertility status was noted [97]. Additional larger-scale studies are needed to assess the effect on infertility.

The use of gonadotropin hormone releasing hormone (GnRh) analogs for 6–12 months post-operatively may help suppress ectopic endometrial activity and provide time for pleural adhesions to create effective pleurodesis [95]. GnRh agonists have been found to be the most effective medical option in preventing the recurrence of catamenial chest pain for as much as 1 year post operatively [98]. Although effective, GnRh agonists may cause menopausal symptoms and osteoporosis, limiting their long-term use. Add-back therapy with progesterone mitigates some of the hypoestrogenic effects. In a study of seven centers, a total of 50 women were evaluated for recurrence of CP based on post-operative treatment with oral contraceptives, GnRh agonist, or no hormonal treatment; patients treated with a GnRh agonist following a surgical intervention had a recurrence rate of 3.4%, versus 83.3% on no hormonal treatment, and 40% on oral contraceptives [99]. The mean follow-up was 39 months, and the surgical interventions varied. Tsuboshima et al. published a recent retrospective observational study with 248 patients, wherein the post-operative group who did not receive hormonal treatment had a 32.6% recurrence rate of pneumothorax, compared to 12.9% in patients who did receive treatment. The most common treatment reported was dienogest (in 56%); the duration of treatment was not reported, but the follow-up period was 832 days [50]. Dienogest, a fourth-generation oral progestin, may be associated with menstrual irregularities and a higher risk of thromboembolic events [17]. Of note, Ciriaco et al. showed that estrogen–progesterone pills alone were not an effective post-surgical treatment regimen, as the cohort placed on this treatment had a 100% catamenial pneumothorax recurrence rate [17].

Fukuda et al. found a higher recurrence rate following surgery in patients with symptoms of catamenial pneumothorax when compared to catamenial hemoptysis [24]. Patients treated for hemoptysis seem to respond to hormonal therapy and have decreased recurrent rates [31]. In a systematic review of nine studies involving 31 patients, Andres et al. reported that there were no recurrences after surgical or medical treatment of lung endometriosis during the follow-up of 22.6 months [9].

## 7. Conclusions

Thoracic endometriosis syndrome should be suspected in any woman of reproductive age who present with persistent respiratory symptoms temporally related to menses [75]. TES is an often debilitating, relapsing and remitting manifestation of endometriosis. The presence of severe pelvic endometriosis is often noted [17,23,45,68,70]. The risk factors remain unclear, and the recurrence rate is relatively high. Every patient who presents for endometriosis consultation should be asked about extra-pelvic symptoms [81]. The patient should be managed conservatively as much as possible; however, if surgery is indicated, a chest and abdomen MRI should be considered [17]. Referrals to a large multidisciplinary team at a tertiary referral center, at a regional or national level, should be attempted [16,100]. The goal of VATS and video laparoscopy is the meticulous inspection and excision of all visible endometriotic implants with and without robotic assistance [8]. Post-operatively, a joint follow-up with the thoracic surgeon and gynecologist is recommended. Selective pleurodesis and hormonal therapy postoperatively appear to decrease recurrence. There is a paucity of data surrounding long-term management and outcomes. There is a need to standardize the surgical approach and post-operative hormonal therapy [17,58]. The creation of a registry could help us better understand and treat the disease [25,31].

### 7.1. CASE 1: Thoracic Management of TES Without Concurrent Gynecology Evaluation

A 40-year-old patient was referred to our office for consultation regarding evaluation and management of endometriosis and infertility. She was initially referred due to the suspicion of endometriosis contributing to her egg quality. She reported a 20-year history of dysmenorrhea and heavy menstrual bleeding. In 2018, she experienced a spontaneous right pneumothorax (no tension and no suspected blebs) after suffering from a chronic cough exacerbated by wildfires. She was admitted for 3 days of conservative management, and had spontaneous resolution without a chest tube. In 2020, she discussed her abnormal uterine bleeding and dysmenorrhea with two separate gynecologists, and was dismissed due to a normal pelvic ultrasound result. In 2020, she experienced chest pain, dyspnea and cough, and was diagnosed with a right pneumothorax, hemothorax and diaphragmatic perforation. She underwent VATS, diaphragm repair (without mesh), and mechanical pleurodesis. It was at that time that she was diagnosed with endometriosis of the chest, lung and diaphragm. She was started on norethindrone acetate 5 mg daily, and underwent pelvic MRI that showed extensive endometriosis. Several months later, in 2021, she had a stable post-operative right pneumothorax, where her chest CT showed a small residual right pneumothorax with small fluid collection, and she was recommended for VATS again. She underwent right VATS decortication, upper-lobe wedge resection, middle-lobe wedge resection, lower-lobe wedge resection, and mechanical pleurodesis. During this admission, norethindrone acetate was increased to 5 mg twice daily. After this, her pelvic pain subsided and her condition improved. Subsequent MRI of the pelvis showed regression of adenomyosis and endometriotic lesions likely due to the hormonal therapy. In 2022, she started IVF (in vitro fertilization), which resulted in two failed embryo transfers and one 20-week miscarriage. During her recent consultation, her ultrasound examination showed a 64 × 51 mm uterus, suspected rectovaginal septum endometriosis, adenomyosis, and thickened uterosacral ligaments. Her right ovary contained a complex endometrioma 54 × 37 mm, with a possible hematosalpinx. Her left ovary contained a 33 × 22 mm endometrioma. She is scheduled to undergo a video laparoscopy for treatment for endometriosis at the time of this publication.

### 7.2. CASE 2: Surgical Management of TES, with the Use of Mesh

**Figure 3:**
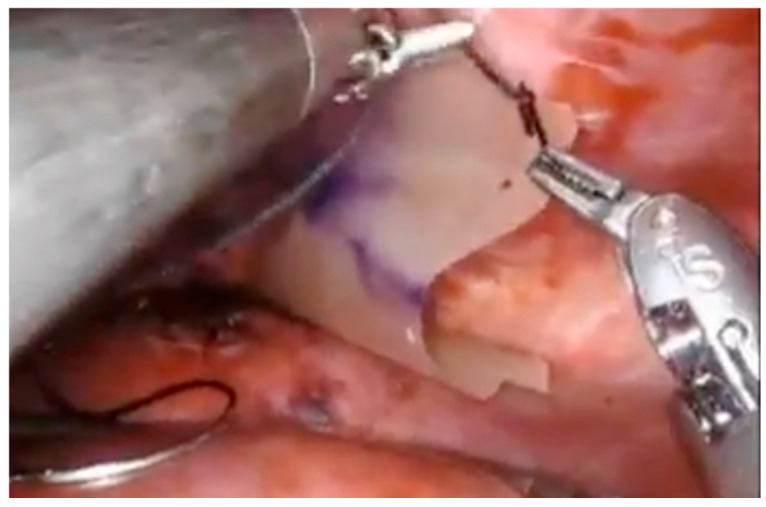
Endometriosis of the diaphragm, treatment with mesh.

This video shows the excision of endometriosis of the diaphragm with mesh placement in a 37-year-old female with a long-standing history of endometriosis and hemothorax, and with a past medical history of VATS and pleurodesis (Figure 3). She suffered from recurrent hemoptysis. She desired conservative surgery to preserve her childbearing potential. Hormonal suppressive therapy helped for 2 months, but her symptoms recurred. Following the surgical resection of endometriosis of the diaphragm and thorax, she has been asymptomatic.

Click the link to see full video
